# Anesthesia for Bronchoscopy—An Update

**DOI:** 10.3390/jcm13216471

**Published:** 2024-10-29

**Authors:** Basavana Goudra, Lalitha Sundararaman, Prarthna Chandar, Michael Green

**Affiliations:** 1 Department of Anesthesiology and Perioperative Medicine, Thomas Jefferson University, Philadelphia, PA 19107, USA; 2 Sidney Kimmel Medical College, 111 S 11th Street, #8280, Philadelphia, PA 19107, USA; 3 Department of Anesthesiology, Brigham and Women’s Hospital, 75 Francis St., Boston, MA 02115, USA; 4 Department of Pulmonary, Allergy and Critical Care, Thomas Jefferson University, Philadelphia, PA 19107, USA

**Keywords:** bronchoscopy, remimazolam, dexmedetomidine, navigational bronchoscopy, endobronchial valve, thermoplasty

## Abstract

The field of interventional pulmonology has grown immensely and is increasingly recognized as a subspecialty. The new procedures introduced in the last decade pose unique challenges, and anesthesiologists need to readapt to their specific demands. In this review, we extensively discuss the pathophysiology, technical aspects, preprocedural preparation, anesthetic management, and postprocedural challenges of many new procedures such as navigational bronchoscopy, endobronchial valve deployment, and bronchial thermoplasty. Majority of these procedures are performed under general anesthesia with an endotracheal tube. Total intravenous anesthesia with rocuronium as a muscle relaxant seems to be the standard US practice. The easy availability and proven safety and efficacy of sugammadex as a reversal agent of rocuronium has decreased the need for high-dose remifentanil as an agent to avoid muscle relaxants. Additional research is available with regard to the utility of nebulized lidocaine and is discussed. Finally, two newer drugs administered for conscious sedation (typically without the need of an anesthesiologist) are likely to gain popularity in the future. Remimazolam is a new short-acting benzodiazepine with a relatively faster offset of clinical effects. Dexmedetomidine, a selective adrenergic agonist, is increasingly employed in bronchoscopy as a sedative during bronchoscopic procedures.

## 1. Background

Interventional bronchoscopy is a promising and expanding branch of medicine. Until recently, the field was largely limited to procedures like bronchoalveolar lavage, sputum collection, foreign body removal, the placement of stents, and endobronchial ultrasound. However, recently, many new procedures have been added to the armamentarium, such as robot-assisted bronchoscopy and the deployment of endobronchial valves. Some newer procedures like bronchial thermoplasty (BT) are taking a backseat. The most recent addition to these procedures is catheter ablation for lung cancer. In addition, developments have taken place in anesthesia too; one of the most noticeable of these is in the field of pharmacology. Sugammadex, a new skeletal muscle relaxant reversal agent, has largely eliminated the need for high-dose remifentanil based total intravenous anesthesia. Pulmonologists are more recently drawn to a newer benzodiazepine, remimazolam, a faster-acting version of midazolam (versed) with unique properties suited to certain bronchoscopy procedures such as bronchial lavage and flexible diagnostic bronchoscopy. Another drug gaining popularity during bronchoscopy is dexmedetomidine. Although both were discussed briefly in the previous review, an updated discussion is warranted. The use of laryngeal mask airway (LMA) has diminished, and many pulmonologists are comfortable with a larger-than-“standard” endotracheal tube for procedures such as endobronchial ultrasound. In this review, we aim to address contemporary anesthesia management of both mainstream and newer bronchoscopic procedures.

## 2. Role of Local Anesthetics in Bronchoscopy

With the increasing popularity of deep sedation and general anesthesia for the majority of bronchoscopic procedures, the role of local anesthesia as a sole anesthetic has vastly diminished. Even for routine diagnostic bronchoscopy, at Jefferson, we use moderate sedation. However, in certain high-risk patients such as those on high-flow nasal cannulas or those with very high oxygen requirements, local anesthesia alone might be safer. Local anesthesia is, however, used frequently along with sedation.

However, nebulized lidocaine continues to be employed in some countries and certain situations such as diagnostic flexible bronchoscopy to suppress coughing. Recently, more studies have been published on the mode of delivery.

Dhooria et al. (2020) conducted study on the optimum mode of delivering lidocaine [1]. In a total of 1050 subjects (randomized 1:1:1), they compared nebulized lignocaine (2.5 mL of 4% solution), oropharyngeal spray (10 actuations of 10% lignocaine), and combined nebulization (2.5 mL, 4% lignocaine) with two actuations of 10% lignocaine spray. Their primary end point was a comparison of the severity of coughing as rated by the patient. Cough severity as rated by the bronchoscopist and procedural satisfaction were other measures. They concluded that, for diagnostic flexible bronchoscopy, ten actuations of 10% lignocaine oropharyngeal spray provided the best conditions. However, it should be noted that, in addition to the above, all patients also received 5 mL of lignocaine gel (applied to the nasal cavity) and four aliquots of 2 mL of 1% lignocaine solution. The total lidocaine dose on average in each group was about 300 mg. In another study, Islamitabar et al. (2022) compared nebulized lidocaine and intratracheally administered (spray-as-you-go) lidocaine for their ability to reduce pain and cough during bronchoscopy [2]. They concluded that nebulized lidocaine was more effective in both cough and pain reduction. It should be noted that, often, sedatives such as fentanyl and midazolam are administered along with local lidocaine during flexible bronchoscopy. In patients undergoing bronchoscopy requiring endobronchial or transbronchial biopsy, Dreher et al. (2016) found that nebulized lidocaine was both safe and effective [3]. In addition, when compared to the lidocaine spray technique, the nebulized group required less lidocaine and fentanyl.

Lidocaine is also administered by the intravenous route to suppress the cardiovascular response to endotracheal intubation (such as elevated blood pressure and pulse) and other responses such as cough reflexes, occasional dysrhythmias, increased intracranial pressure, and increased intraocular pressure. This is especially true in certain patient populations like those with atherosclerotic heart disease, potential intracranial lesions, and potential penetrating eye injuries [4]. It can effectively blunt cough reflexes and dysrhythmias [5]. In patients with chronic obstructive pulmonary disease undergoing bronchoscopy, intravenous lidocaine infusion resulted in a significant reduction in propofol requirements [6]. Patients in this study received 2 mg/kg followed by 4 mg/kg/h of lidocaine in addition to propofol infusion.

A word of caution is warranted here. It is generally accepted that major adverse events related to lidocaine are seen at plasma levels above 5 mcg/mL [7], and serious toxicity such as seizures and fatal cardiac arrhythmias occur only at 3 to 4 times such concentrations. Nonetheless, specific groups such as elderly people and those with long hospitalizations, congestive heart failure, and diminished hepatic clearance of lidocaine are predisposed to serious adverse reactions [8,9,10] at doses considered as safe in healthy individuals. It is reported that administration of a 280 mg dose via intermittent positive pressure breathing and nebulization of a 400 mg dose via ultrasound resulted in plasma concentrations of lidocaine no more than 1.1 µg/mL [11]. Contrarily, seizure was reported in a 28-year-old with AIDS, chronic end-stage renal failure, anemia, congestive heart failure (CHF), cardiomyopathy, and increased liver function tests, after administration of a total dose of topical lidocaine (300 mg). Plasma lidocaine concentrations in this patient, soon after seizure and at 4 and 22 h, were 12.0, 7.6, and 1.4 mg/L, respectively. As a result, it is important to review patients’ co-morbidities with a particular reference to cardiac and liver function.

## 3. Electromagnetic Bronchoscopy Techniques and Their Anesthetic Implications

### 3.1. History, Methodology, and Indications

While respiratory infections continue to be the most common indication for flexible fiberoptic bronchoscopy, lung nodules are the second most common indication [12]. In both men and women, lung cancer is the second most common cancer [13]. Low-dose computed tomography is currently recommended for lung cancer screening [14]. Patients subjected to such screening are typically elderly with a significant smoking history.

As a group, they clearly pose a higher anesthesia risk. Beyond that, the low-dose CT scan employed as a screening tool has limitations. The US Preventive Services Task Force finds that LDCT has a sensitivity that ranges from 59% to 100%, specificity of 26.4% to 99.7%, positive predictive value of 3.3% to 43.5%, and negative predictive value of 97.7% to 100%. By definition, a lung nodule is less than three centimeters in size. Only about 5% of small nodules are cancerous and the remainder could be granulomas (benign) tumors or cysts, inflammatory diseases, scar tissue from an old infection, or congenital lung abnormalities. The risk of malignancy increases with the size of these modules. Only nodules that are considered as high-risk or seen to increase in size in follow-up scans may require biopsy.

A major challenge with any technique of bronchoscopy is to achieve a high diagnostic yield of the lesion in question. Navigational bronchoscopy (NB), the most recent form being robot-assisted NB, is a novel technique uniquely suited for diagnosing both peripheral and central nodules. Some use electromagnetic fields, while others might use shape sensing technology. It has the best combination of higher diagnostic yield and a low complication rate [15]. It involves obtaining a chest CT before the procedure and creating a three-dimensional (3D) virtual airway map through which the bronchoscopist navigates to locate the lung nodules. This is followed by biopsy and (or) interventions such as the use of a fiducial marker before resection [15].

Regardless of the technology employed to access the peripheral pulmonary lesions, in addition to many factors such as lesion location, lesion size, the presence of a bronchus sign, and registration error, anesthesia strategy is important. The majority of anesthesiologists work in the bronchoscopy suite on an ad hoc basis and, as a result, might lack an understanding of the procedural aspects and consequent anesthesia implications [16].

The anesthesiologist should do everything to provide the most optimum conditions for the pulmonologist to reach the lesion and obtain a biopsy. The CT scans, on which the NB relies, are performed in patients who are awake and breathing air. Pulmonary atelectasis is a form of body divergence in computed tomography and can obscure the target lesion in intraprocedural cone beam computed tomography images, thereby affecting both the navigational and diagnostic yield of the procedure [17]. However, in contemporary practice, most places (including at Jefferson) do not use cone beam computed tomography; most rely on conventional fluoroscopy or augmented fluoroscopy. Performing a staging endobronchial ultrasound before NB poses further challenges. Administration of general anesthesia, both intravenous and inhalational, causes lung collapse, which is seen in about 90% of all patients [18]. It can also result in false-positive radial probe ultrasound images. In patients undergoing bronchoscopy, 89% of patients were found to have atelectasis in one segment, while 32% experienced this in at least six of the eight evaluated segments [17,19]. In this context, the anesthesia goals/requirements for NB include avoidance of excessive chest movement and avoidance of atelectasis.

Park P et al. (2021) measured postoperative atelectasis with an ultrasound 30 min after the procedure and transfer to the postoperative care unit. Those administered an inspired oxygen of 0.35 during induction and recovery experienced significantly less atelectasis than those who received 0.6 [20]. Similarly, Ashraf M et al. studied 60 patients undergoing laparoscopic cholecystectomy, who received either 40% oxygen or 90% inspired oxygen, both after endotracheal intubation and for 2 h postoperatively; 60% in the first group and 76.7% in the second had atelectasis as seen on a computed tomography scan [21]. All patients had volume-controlled ventilation with zero positive end-expiratory pressure (PEEP). However, Jiang et al. (2023), who randomized 120 patients into two groups who received either 30% or 60% FiO_2_ (fraction of inspired oxygen) during mechanical ventilation in a 1:1 ratio [22], did not find any difference in the degree of atelectasis. It should be noted that none of their patients received PEEP or were administered any recruitment maneuvers.

The mechanism of atelectasis is three-fold and these are discussed in a review [23]. The first is the reduction in transmural pressure that keeps the alveolus open. Induction of anesthesia results in diaphragm relaxation and its caudal displacement, which removes the differential pressures in the abdomen and chest. The resulting compression atelectasis is most prominent in the dependent lung regions. The second mechanism is gas absorption. It can be caused by airway obstruction and distal air absorption and/or faster absorption of oxygen in those alveoli where perfusion is high in relation to ventilation. If these alveoli are ventilated with a high percentage of oxygen, the effect is greater. The third mechanism is the impairment of pulmonary surfactants. If a bronchoscopist performs a lavage before NB, the saline washes away the surfactant. Excessive suctioning of the bronchus is another factor contributing to atelectasis. It is important to keep suctioning and any possible trauma to the minimum. The degree of atelectasis is less in patients with chronic obstructive lung disease.

### 3.2. Type of Anesthesia

General anesthesia is the standard for patients undergoing NB. Both total intravenous anesthesia and inhalational anesthesia are appropriate, and both are shown to cause atelectasis. Unlike endobronchial ultrasound, where the preferred airway is an LMA, patients undergoing NB typically receive an endotracheal tube, usually 1 mm larger in diameter than normally used(8.5), if the laryngeal opening allows. An ETT allows the application of PEEP to preserve the lung volume and the larger bore enables the procedure’s performance without compromising the airway area significantly. During anesthesia induction, if it can be accomplished safely, one should avoid 100% oxygen administration to limit rapid development of atelectasis. Edmark et al. looked at atelectasis in patients undergoing induction with 100, 80, and 60% inspired oxygen [24]. Immediately after the period of apnea, the CT scan showed that the mean atelectasis area in the basal scan was close to 10 cm^2^ (5.6%) in the 100% group compared to 1.3 cm^2^ (0.6%) and 0.3 cm^2^ (0.2%) in the 80 and 60% groups. Considering that there was no difference between the 80 and 60% groups, 80% oxygenation seems to be appropriate. For maintenance of anesthesia, one can use air/nitrogen as part of the gas mixture, which will limit the early formation of atelectasis. The performance of a vital capacity maneuver followed by the administration of a nitrogen mixture assists in restoring the lung volume. Ventilation with high concentrations of oxygen leads to the quick reappearance of atelectasis and should be avoided.

### 3.3. Ventilatory Strategy

A ventilatory strategy to prevent atelectasis (VESPA) is employed at Thomas Jefferson hospital, Philadelphia, USA, and the following are its components [17,25]: volume control, tidal volume (TV) of 6–8 cc/kg of ideal body weight (IBW), administering the lowest tolerable inspired oxygen to aim for a saturation of about 94%, a PEEP of 8–10 cmH_2_O, and the performance of a recruitment maneuver immediately after intubation (10 consecutive breaths at a plateau pressure of 40 cmH_2_O, with a PEEP of 20 cmH_2_O in pressure control mode).

A second strategy that aims to limit atelectasis and excessive ventilatory chest excursions with an aim to avoid computed tomography-to-body divergence is the lung navigation ventilation protocol (LNVP) [26]. The protocol employs a dual ventilation strategy with pressure-controlled continuous mechanical ventilation and patient-specific VT at 10–12 cc/kg of IBW. Like VESPA, it recommended to use the minimum tolerable inspired oxygen, with a PEEP of 10–15 cmH_2_O (for upper/middle lobe lesions) and 15–20 cmH_2_O (for lower lobe). The recommended post-intubation recruitment consists of four hand-delivered breaths with 30 cmH_2_O over 30 s or 40 cmH_2_O over 40 s [17]. Any experienced anesthesiologist understands that it is difficult to accurately replicate these recommendations. LNVP is shown to have a better diagnostic yield (92% vs. 70%), although this is statistically not significant [26].

Nevertheless, one should be mindful of the barotrauma and hemodynamic instability the recruitment maneuvers can cause. At least in animal models, it is shown that a higher margin of safety was obtained when a higher PEEP and lower driving pressure strategy was used for recruiting the lung [27]. The addition of periodic sighs is also shown to recruit the lungs better [28]. Hypotension is reported in 79.8% of 94 American Society of Anesthesiologists physical status I–II patients, aged 19 to 75, with scheduled spinal surgery [29].

Grant et al. (2024) studied the contribution of atelectasis to CT-to-body divergence (described as the difference between preprocedural CT scans and intraprocedural lung architecture) [30]. They correctly recognized that standardized PEEP levels may not be appropriate for all patients due to hemodynamic and ventilatory instability. They evaluated whether incremental increases in PEEP resolved atelectasis, as demonstrated by a transition from a non-aerated pattern to an aerated appearance on radial probe endobronchial ultrasound (RP-EBUS) and concluded that RP-EBUS is indeed an effective tool to monitor the resolution of atelectasis within a lung segment with increasing levels of PEEP.

### 3.4. Patient Positioning

Attention was drawn to positioning-related atelectasis under general anesthesia with mechanical ventilation by Klingstedt et al. (1990) [31]. After induction in the supine position, the cross-sectional area for both lungs was reduced in four out of five patients. Application of PEEP reduced but did not eliminate the atelectatic areas. Lateral positioning was similarly associated with atelectasis mainly in the dependent lung.

Paralysis and positive pressure ventilation are standard for patients undergoing NB under general anesthesia. Maintenance could be achieved with propofol-remifentanil as part of total intravenous anesthesia (TIVA) or inhalational anesthesia. At Jefferson, we typically use inhalational anesthesia along with low-dose propofol infusion, with rocuronium as a muscle relaxant. If the patient is appropriate for reversal and extubation, we administer sugammadex as a reversal agent.

### 3.5. Perioperative Complications

One of the perioperative complications of NB, although infrequent, is pneumothorax is. In their retrospective analysis, Mwesigwa et al. (2024) documented a pneumothorax rate from 3.4% (in the 2022–2023 period) to 9.8% (in the 2020–2021 period). Clearly, in their analysis, the rates came down as they gained experience. In addition, the majority of them were in the upper lobe regions [15]. In another prospective study, the investigators observed a pneumothorax rate of 4.3%, a serious bleeding rate of 1.5%, and a respiratory failure rate of 0.4% [32]. In total, 2.9% (35 of 1215) of patients experienced pneumothorax of sufficient severity (Common Terminology Criteria for Adverse Events scale grade 2 or greater) requiring hospitalization or intervention. However, these rates are significantly less than percutaneous techniques (about 26%) [33].

## 4. Anesthesia and Deployment of Endobronchial Valves

### 4.1. History, Pathophysiology, and Indications

Among the many factors that contribute to shortness of breath (SOB) and poor exercise tolerance in patients with emphysema, hyperinflation, especially dynamic hyperinflation, plays a central role [34]. There is a reduction in lung recoil caused by damage to the elastic fibers, while the chest wall compliance is preserved. The end-expiratory lung volume (EELV), functional residual capacity (FRC) and total lung capacity (TLC) increase [35]. There is also increased air trapping (as a result of small airway closure in the dependent lung areas) and [36] dynamic hyperinflation that results from mismatch of the expiratory time constant of the lung and time between consecutive breaths. This especially manifests during exercise because there is less time for expiration, which results in more air trapping. The exertion of breathing increases, which will eventually lead to respiratory failure. Any superadded pulmonary infection will cause acute worsening of symptoms.

Surgical lung volume reduction was described in 1959 by Brantigan et al. and before that by Crenshaw et al. [37,38]. Although there was clinical improvement in one-fourth of these patients, operative mortality was excessive (18%). Cooper et al. described an improvement in the technique with reduced mortality [39].

Nevertheless, a non-surgical approach, in the form of deploying one-way endobronchial valves, is becoming more popular [40,41,42]. In properly selected patients, it aims to replicate the results of lung volume reduction surgery without the associated morbidity and mortality. Emphysema patients with severe hyperinflation with a suitable lobe that can be rendered atelectatic without collateral ventilation are appropriate candidates [40]. The inclusion and exclusion criteria for endobronchial valves largely evolved from the National Emphysema Treatment Trial (NETT) [43]; the LIBERATE trial, the first multicenter randomized controlled trial to evaluate the effectiveness and safety of the Zephyr Endobronchial Valve (EBV) in the USA in patients with little to no collateral ventilation, paved the way for FDA approval in Jan 2018 [42,44,45,46,47]. In addition, both zephyr and spiration are employed for persistent air leaks.

### 4.2. Anesthesia Aspects

These procedures are performed either under deep sedation or, preferably, under general endotracheal anesthesia. Regardless of the type of anesthesia, evaluation for the appropriateness of endobronchial valve deployment by using a catheter connected to a Chartis console is required. The console measures the air flow and pressure from the occluded lobe and quantifies the collateral ventilation status [48,49,50,51].

At Jefferson health, Philadelphia, we perform these procedures under general anesthesia with an endotracheal tube (ETT). Anesthesia induction and maintenance is achieved with intravenously administered drugs, predominantly propofol and remifentanil in the form of TIVA. When performed under GA, Chartis measurements are found to be shorter, with no difference in target lobe volume reduction following EBV treatment [52].

Direct anesthesia-related complications are uncommon. Hypotension is to be expected and phenylephrine infusion (titrated as necessary) is often required.

Both chest pain (non-cardiac origin) and pneumothorax occur frequently. Chest pain occurs more frequently with deep sedation than general anesthesia (40% vs. 18%). In the same study, pneumothorax occurred in 24% of patients under sedation vs. 33% under general anesthesia [53]. The incidence is reported as 4.2–34.4% [47,53]. A potential drawback of general anesthesia is the increased likelihood of false-positive interlobar collateral ventilation due to use of positive pressure ventilation. Before the deployment of the last two valves, the bronchoscopist usually asks for lower PEEP and low tidal volume (TV reduction of 20–25%).

#### 4.2.1. Mechanism and Management of Pneumothorax

**Figure 1:**
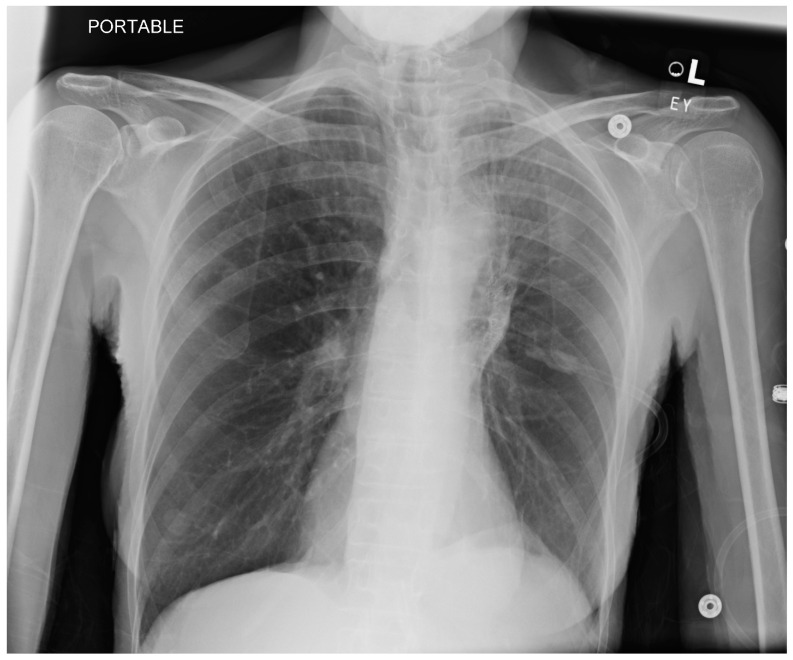
Pre-procedure chest X-ray (CXR): Pt is a former smoker, who smoked 30 cigarettes a day for 15 years and quit 20 years ago, with severe COPD and deemed suitable for bronchial valve. L—indicated left side, EY—the initials of the radiographer, the dots in the 3 squares are electrocardiogram electrodes.

**Figure 2:**
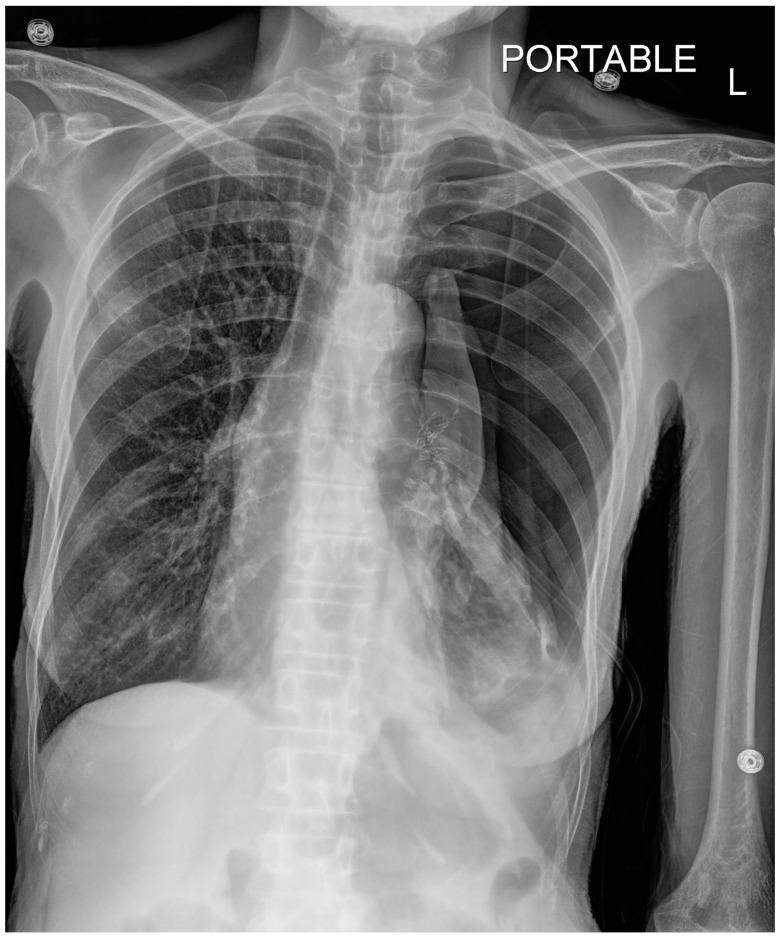
CXR, taken about 16 h after placement of bronchial valve. Large left pneumothorax with rightward mediastinal shift. Left basilar atelectasis. L—indicated left side.

**Figure 3:**
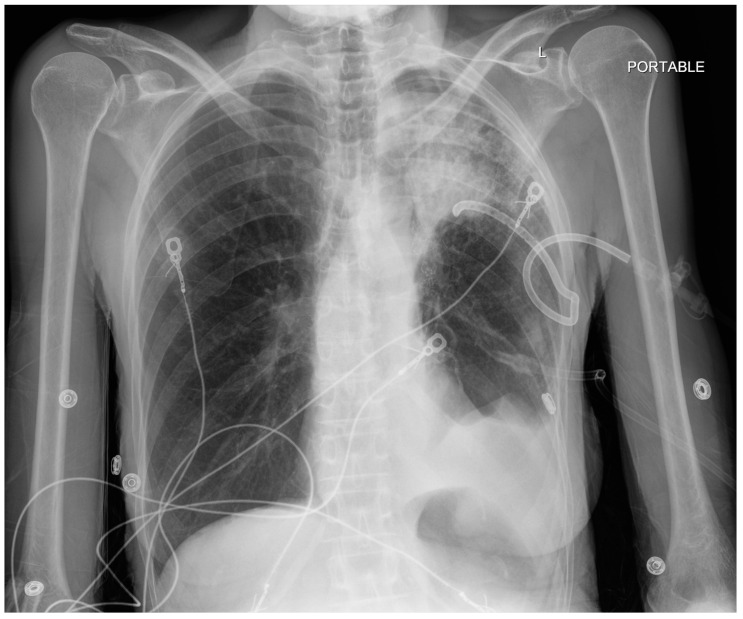
CXR interval placement of a left chest tube. Likely residual loculated pneumothorax at the left lung base. Left upper lobe opacification. L—indicated left side.

**Figure 4:**
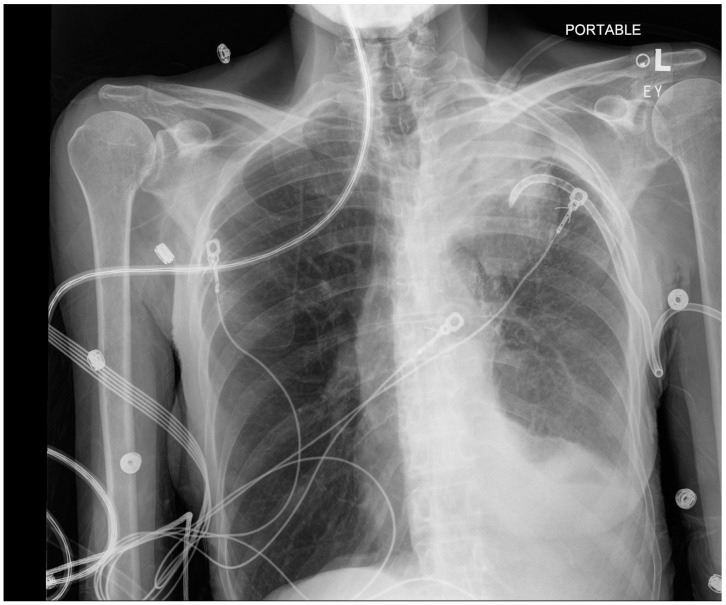
CXR left upper lobe volume loss (day 2). Moderate left pleural effusion compatible with hemothorax (which was seen on CT scan). Stable cardiac silhouette. Left apical chest tube. Left hilar endobronchial valves. L—indicates left side, EY—the initials of the radiographer.

**Figure 5:**
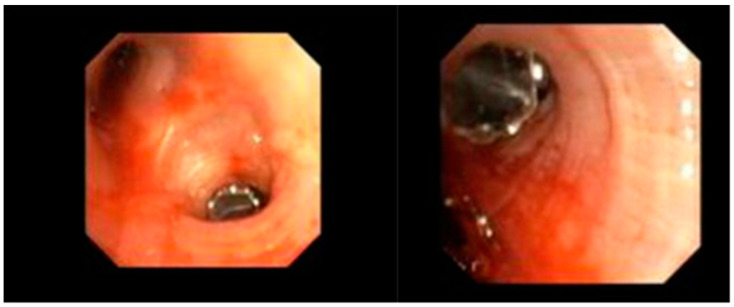
Bronchial valve placed (size 4, left upper lobe).

Pneumothorax (Figure 1, Figure 2, Figure 3 and Figure 4) developing after the placement of the endobronchial valve (Figure 5) is usually managed by pulmonologists; however, anesthesiologists should be aware. Dijk et al. published their revised expert statement that addresses the issue of pneumothorax extensively [54]. The development of pneumothorax is related to compensatory expansion of the untreated ipsilateral lobe. Such an expansion might result in the rupture of blebs, bullae, and fragile lung tissue [55]. The bronchopleural fistula that develops leads to air leak, which can become worse and clinically significant very quickly. Pneumothorax can also develop in the vacuum created by therapeutic lung collapse (pneumothorax ex vacuo). The air enters the potential space from the ambient tissues and blood [56]. As there is no bronchoalveolar fistula in this situation, a chest drain is not necessary, and the pneumothorax will spontaneously resolve over time.

Pneumothorax can develop in the immediate postoperative period, in the post-anesthesia care unit, or within the first 3 days post-anesthesia [47,57]. Valipou et al. published their management algorithm for pneumothorax [58]. Nearly 80% of them happen in the first 48 h, with 10% in about 3–5 days and 10% after day 6 [59,60]. Both anesthesiologists and bronchoscopists should be particularly vigilant about the development of tension pneumothorax. Certain post-procedural protocols (cough suppression, strict bed rest, not letting the patient elevate their arm above their shoulders) are employed at Jefferson to minimize the pneumothorax risk. Most pneumothoraxes are treated conservatively with serial imaging; some may require chest tubes and, rarely, valve removal.

#### 4.2.2. COPD Exacerbation

COPD exacerbation was reported in three of the twenty endobronchial valve insertions performed at the Queen Elizabeth Hospital, Woodville, South Australia, in 2018. Ten patients received monitored anesthesia care, while the remaining 10 received general anesthesia with an endotracheal tube [61]. All patients had their Chartis measurement and EBV implantation performed in one sitting. Chartis measurements took slightly longer in patients receiving monitored anesthesia care. The authors preferred total intravenous anesthesia, which has the benefit of preserving hypoxic pulmonary vasoconstriction.

In a retrospective chart review of 202 procedures, of which 198 were performed under general anesthesia with ETT and the remaining with an LMA, hypotension was the most commonly observed intraprocedural adverse event [62]. While one patient sustained severe hypotension, the remainder were considered significant or moderate. Noradrenaline, phenylephrine, and/or ephedrine were administered in most of the procedures. This was followed by desaturation. There were no deaths or unplanned ICU admissions. None of the patients required cardiopulmonary resuscitation or reintubation [62]. Other complications mentioned in the literature are acute bronchitis, pneumonia, and/or lung infections developing in the first 3 months of the procedure. They are unlikely to concern the anesthesiologist [63,64].

## 5. Bronchial Thermoplasty

### 5.1. Preoperative Concerns and Indications

In the past few decades, radiofrequency (RF) ablation has been used as a therapeutic tool in many settings. The technique uses RF energy to produce heat and destroy the target tissue. The technology has been in use for treating cardiac arrhythmias for a long time and more recently for the treatment of type 2 diabetes. BT uses RF ablation to impact airway remodeling, including a reduction in excessive airway smooth muscle within the airway wall [65]. It is employed in the treatment of severe asthma and involves RF energy delivery to the larger airways in more than one sitting. It is presumed to reduce the airway smooth muscle, which is responsible for bronchospasm. The treatment has led to improvements in asthma control and quality of life [66].

RF ablation should be offered only to patients with documented asthma. Some of the warnings and precautions include chronic obstructive pulmonary disease, bronchiectasis, recurrent respiratory infections, or any other uncontrolled significant respiratory disease [67]. RF thermal energy is typically delivered to the airway wall, as part of a series of three separate bronchoscopy sessions at least 3 weeks apart. The FDA approved this treatment modality on 27 April 2010 for those with severe persistent asthma, aged ≥ 18 years, and whose asthma is not well controlled with inhaled corticosteroids and long-acting β_2_-agonists. Post-FDA-approval trial results are now available. Chupp et al. published their 5-year experience in 2021. The study involved 284 patients that were enrolled at 27 centers [68]. Their results indicated that treated patients experienced decreases in severe exacerbations, hospitalizations, ED visits, and corticosteroid exposure. They concluded that BT improves asthma control in different asthma phenotypes. Wijsman et al. found downregulation of the gene expression of airway epithelium related to the airway inflammation gene set in those treated with BT [69].

### 5.2. Anesthesia Considerations

It is recommended that to undergo BT, the patient should be symptomatic (stable asthma) for 48 h, without active respiratory tract infection and with no acute exacerbation of asthma for 2 weeks before BT [70,71]. BT can be performed under topical anesthesia with sedation or general anesthesia. A relatively large cohort of 13 severe asthma patients underwent successful RF ablation under moderate target-controlled infusion (TCI) propofol/remifentanil sedation [72]. Both patients and bronchoscopists reported high satisfaction.

However, many bronchoscopists prefer general anesthesia. Case reports, case series, and a prospective cohort trial detailing the anesthesia experience and recommendations have become available [71,72,73,74]. Anesthesiologists expect these patients to have poorly controlled asthma with abnormal pulmonary function tests. In preparation for the procedure, pulmonologists routinely administer steroids. The recommended dose is 50 mg/day of prednisolone or equivalent for 5 days, starting treatment 3 days prior to the procedure [65]. These patients typically present to the hospital on the day of the procedure.

Both deep sedation without a definitive airway and general anesthesia with an ETT of an LMA have been used. Our experience is limited to general anesthesia with an ETT and a TIVA technique. An anticholinergic such as glycopyrrolate may be administered before induction, for its antisialagogue properties. However, it can interfere with clearing secretions post bronchoscopy and may produce tachyarrhythmias. Administration of nebulized salbutamol and ipratropium is beneficial.

Induction is generally performed with fentanyl and propofol. However, remifentanil or alfentanil are good options too. Rocuronium is an appropriate muscle relaxant, regardless of the airway planned (ETT or an LMA). A potential downside of an ETT is airway irritation and worsening of bronchospasm. Nevertheless, the procedure itself causes tracheobronchial irritation. Maintenance of anesthesia is generally achieved with propofol and remifentanil. Any coughing during the procedure needs to be abolished, and to this end, it is important to monitor the neuromuscular junction. Intermittent positive pressure ventilation (IPPV) is instituted via a swivel connector to secure an airtight seal while a flexible bronchoscope is inserted. The connector also provides 360 degrees of rotation. The RF energy is delivered via a catheter introduced through the flexible bronchoscope to the desired bronchial location, in a distal to proximal direction. These procedures can take about 45–60 min. Muscle relaxant reversal is achieved with sugammadex.

#### Perioperative Complications

Bronchospasm can occur during bronchoscopy and can be managed by delivering salbutamol through the working channel of flexible video bronchoscope. Coughing and wheezing is common after the procedure and salbutamol-steroid nebulization may be sufficient.

Some of the reported complications are hypoxemia, bronchospasm, laryngospasm, atelectasis due to fibrin plugs, exacerbation of asthma, lower respiratory tract infection, and bronchial artery pseudoaneurysms [74,75]. Bronchial artery pseudoaneurysm caused mediastinal hematoma and hemothorax in a 66-year-old woman several days after an uneventful bronchial thermoplasty of the right lower lobe. This patient presented in respiratory distress with diffuse expiratory wheezing, tachycardia, inspiratory crackles of the right lung, and diminished breath sounds at the right base. Right upper lobe pulmonary embolism, right lower lobe consolidation, and pleural effusion with posterior mediastinal involvement were revealed in a chest CT scan. The patient was managed with IPPV in the ICU, and continuing deterioration required the placement of a chest tube (in the setting of an enlarging right pleural effusion and acutely worsening anemia) that was followed by a dramatic improvement in hemodynamics. Finally, embolization of the right bronchial artery was required.

## 6. Depth of Anesthesia Monitoring

Even though all sedatives/anesthetics primarily act on the brain and produce varying degrees of sedation/anesthesia, monitoring their effect on the brain (e.g., depth of anesthesia/sedation) is not the standard of care. Currently, the American Society of Anesthesiologists (ASA) recommends electrocardiography, noninvasive blood pressure, end-tidal carbon dioxide and oxygen saturation as measured by a pulse oximeter as part of minimal mandatory monitoring for all procedures requiring anesthesia. In addition, if general anesthesia is likely to last >30 min, temperature monitoring is required [76]. Even though depth of anesthesia monitoring is known to decrease the incidence of awareness, postoperative delirium, and POCD and improve several postoperative outcomes, it is not widely employed [77].

Bronchoscopic procedures are unique as they do not involve a surgical incision. As a result, the need for opioids is minimum. In addition, they require a relatively “lighter” form of general anesthesia. However, this could become the reason for employing depth of anesthesia monitoring. Erring on the side of lighter general anesthesia would place the patient at higher risk of awareness. With an increase in the use of muscle relaxants, “awake and paralyzed” is a real possibility. In a single-center, prospective, observational cohort study on 383 mechanically ventilated emergency department patients, the prevalence of “awareness with paralysis (AWP)” was 2.6% (10/383). In this study, 70% of those who experienced AWP were exposed to the muscle relaxant rocuronium [78]. The use of rocuronium has become routine for advanced bronchoscopic procedures.

Fadaizadeh et al. enrolled 70 patients undergoing interventional bronchoscopic procedures under midazolam, sufentanil, and propofol, with spontaneous ventilation, and depth of anesthesia monitoring with bispectral index (BIS). In this study, the mean BIS scores were 52 ± 13.5 and 70% of patients had stable BIS between 40 and 60 [79]. BIS scores are electroencephalogram (EEG)-derived dimensionless numbers that provide information on the awareness or sleepy state of the brain. The authors concluded that a mean BIS of 52 ± 13.5 is appropriate for the prevention of complications. Avidan et al. targeted a BIS value between 40 and 60, which is advocated to prevent anesthesia awareness while allowing a reduction in its administration [80]. They randomly assigned 2000 patients to BIS-guided anesthesia or end-tidal (expired anesthesia gas) concentration-guided anesthesia. BIS monitoring, surprisingly, had no impact on the awareness incidence, with two cases of definite anesthesia awareness occurring in each group. As a result, their findings did not support routine BIS monitoring as part of standard practice.

While studying eighteen patients undergoing flexible bronchoscopy with BIS monitoring, Yamada et al. recorded median BIS values at a modified observer’s assessment of alertness and sedation (MOAA/S) score of 3–4. They suggested that a BIS value of 82 reflects an adequate level of sedation [81]. Nevertheless, there is no benefit to using BIS to guide the depth of sedation during propofol sedation for relatively short-duration flexible fiberoptic bronchoscopies [82]. However, total intravenous anesthesia (TIVA) with a muscle relaxant is used more frequently during advanced bronchoscopic procedures and, as a result, the use of EEG brain function monitoring such as BIS or Sedline could be more productive [83]. During rigid bronchoscopy, Bould et al. found that BIS scores were <40 for 99.6% (87.9–100% [0–100%]) for the duration of bronchoscopy [84].

In conclusion, we recommend using an EEG-based depth of anesthesia monitor such as BIS or Sedline for TIVA-based flexible advanced bronchoscopic procedures with a muscle relaxant and during rigid bronchoscopy. Their use is likely to reduce the risk of awareness and minimize the need for anesthetic medications. As a result, patients are likely to be awake sooner. Considering that postoperative pain is rarely a significant issue and that the need for a “back to baseline” awake status is important (for the establishment of effective spontaneous ventilation), it is important to minimize the administration of unnecessary and excess anesthetic medications.

## 7. Ventilation, Oxygenation, and CO_2_ Elimination

The currently recommended monitoring (by the ASA) that includes heart rate and blood pressure, electrocardiogram, a pulse oximeter, and capnography is sufficient for nearly all bronchoscopic procedures. Depending on the procedure, the anesthesia technique employed (flexible vs. rigid and TIVA vs. inhalational anesthesia), anticipated complications (e.g., massive bleeding), and patients’ preoperative physiological status (low ejection fraction, etc.), additional monitoring such as invasive blood pressure monitoring is required.

Both rigid (with or without jet ventilation) and many flexible endoscopic procedures, which are typically performed without a definitive airway (such as an endotracheal tube or an LMA), may pose challenges with regard to monitoring ventilation. It is nearly impossible to obtain a true end-tidal gas sample for accurate monitoring end-tidal carbon dioxide (Et CO_2_).

Measuring carbon dioxide pressure (PCO_2_) transcutaneously (TcPCO_2_) using a sensor probe placed on the earlobe was tried in 74 patients who underwent fiberoptic bronchoscopy [85]. In this study, the mean TcSO_2_ measurement was 95.9 ± 2.27 (80–100%). In addition, patients with a more than 20-packyear history of smoking had significantly higher TcPCO_2_ values compared to the nonsmokers and light smokers. As a result, the authors recommended monitoring PCO_2_ in male patients with endobronchial lesions, those with a longer smoking history, and those with a longer duration of FOB, in addition to SpO_2_. In a prospective observational study that included 60 patients undergoing bronchoscopy with propofol sedation, Yaman et al. found that the highest TcPCO_2_ was 85 mmHg [86]. It is important to measure and titrate sedation appropriately to preserve sufficient spontaneous ventilation so that patients do not experience any highCO_2_-related cardio and cerebrovascular events, especially in those at risk for such events.

A highly significant correlation between arterial CO_2_ and TcPCO_2_ was demonstrated in patients undergoing bronchoscopic lung volume reduction [87]. All the patients had severe chronic obstructive pulmonary disease and were receiving conscious sedation with IV midazolam and IV alfentanil. Monitoring EtCO_2_ is impossible in such situations.

Rigid bronchoscopy is frequently performed under deep general anesthesia and ventilation is facilitated by a jet ventilator. Arterial CO_2_ increases during such procedures if the tidal ventilation is inadequate. If the minute ventilation is held stable, frequency of ventilation (if not too excessive) plays a role too. Ventilation is often monitored by observing chest wall movement and, occasionally, blood gas analysis. The difference between arterial CO_2_ and EtCO_2_ during supraglottic jet ventilation at standard respiratory rate was found to be 13.4 ± 6.8 mm Hg (mean ± SD), while it was 5.7 ± 5.2 mm Hg during conventional ventilation through a standard ETT [88]. Simon et al. found a good correlation between Ert CO_2_ and arterial CO_2_ in patients undergoing rigid bronchoscopy during the steady-state phase and not the dynamic phase [89].

## 8. Management of Lesions High up in the Trachea

In terms of tracheal lesions, patients typically present for dilation with either insertion of a tracheal stent (for tracheal stenosis or extrinsic compression from a mediastinal mass) or debulking (tumors). Common causes of tracheal stenosis include post-intubation or post-tracheostomy injuries. Some of the other causes are autoimmune diseases, infection, gastro-esophageal reflux disease, and radiation injures [90]. The site of the lesion (intra- or extra-thoracic) dictates the shape of the dynamic flow-volume loops that measure forced expiratory and inspiratory vital capacity. The shape of the loop also depends on the level (intra- or extra-thoracic) and the nature (fixed or variable) of the obstruction [91].

### 8.1. Preprocedural Evaluation

Apart from interviewing the patient and explaining the anesthesia, it is important to review the radiology findings (chiefly CT scan) with the pulmonologist. The presence of resting stridor indicates that the internal diameter of the trachea is less than 5–6 mm [92]. Before such a stridor, patients might have experienced exertional dyspnea. These symptoms could suddenly worsen with any inflammatory or infectious condition compromising the tracheal diameter.

### 8.2. Procedural Aspects

Bronchoscopic dilation of stenosis is a safe procedure; however, it carries a higher risk of recurrence. Ntouniadakis et al. compared disease recurrence after CO_2_ laser excisions and balloon dilatation in patients with subglottic stenosis [93]. In this retrospective chart review, 3-year recurrence risk was 73.7% for the CO_2_ laser excisions compared with 51.6% for the balloon dilatation. CO_2_ laser excision procedures were performed under general anesthesia with high-frequency positive pressure ventilation through a steel, laser-resistant catheter. The tissues were either vaporized or divided with radial incisions through suspension microlaryngoscopy. For balloon dilation, they used superimposed high-frequency jet ventilation (HFJV). Absence of a ventilation catheter in the trachea facilitated such an approach. Vernet et al. employed holmium laser treatment for benign tracheal stenosis. The holmium laser eliminated some of the limitations of CO_2_ lasers. These include inadequate hemostasis and visual hindrance with bleeding [94].

### 8.3. Anesthesia Concerns

Total intravenous anesthesia with remifentanil is typically employed during these procedures. The availability of sugammadex has allowed anesthesiologists to employ rocuronium to achieve better procedure conditions; however, maintenance of spontaneous ventilation may be paramount with some tracheal lesions. A discussion with a bronchoscopist is essential to create an appropriate plan. In such cases, patients could be deepened with sevoflurane inhalation, while maintaining spontaneous ventilation. Muscle relaxants will remove the resting muscle tone, which could lead to catastrophic total airway obstruction. Occasionally, patients may be unable to lay supine and might need to be intubated in a sitting position with a fiberscope. In rare instances, depending on the site and severity of stenosis, high airway pressure and severe hypercarbia may develop quickly, and extracorporeal circulation might be lifesaving. In our hospital, we have established femoral–femoral cardiopulmonary bypass prior to induction of anesthesia and intubation above the tracheal tumor orally under general anesthesia, as Zhou et al. reported in their case report [95].

Depending on the co-morbidity and anticipated complications, one might use additional invasive monitoring such as invasive blood pressure monitoring. The presence of an arterial cannula allows samples to be drawn for blood gas analysis. HFJV is frequently employed during anesthesia for tracheal bronchoscopic procedures. It allows the delivery of sufficient oxygen/air mixtures through a narrow tube. The rates vary from 100 to 600/min. The delivered volumes are relatively low, with a short inspiratory time. In fact, the delivered tidal volume is smaller than the dead space and the flow is chiefly laminar [96]. The tip of the catheter could be placed in various locations such as above or below the glottis or in the trachea. Barotrauma (with resultant pneumothorax and subcutaneous emphysema), mucosal injury from the dry gas, or airway obstruction are some of the HFJV-related adverse events. Kinked catheters, bleeding, and surgical emphysema were reported, together at a rate of 20%, in 50 consecutive procedures, for which 44 patients were ventilated with transtracheal HFJV by Anderson et al. [97]. All patients presented with severe airway compromise with stridor and were undergoing pharyngolaryngeal surgery. They employed HFJV at a rate of 100 min^−1^, which was incrementally decreased if the pause pressure exceeded 10 mbar. Their initial ventilator settings were a driving pressure of 1 bar, frequency of 100 min^−1^, inspiratory–expiratory (I:E) ratio of 1:2.5, a pause pressure 10 mbar, and FIO2 1.0. The driving pressure was increased to maintain an SpO_2_ of >95%. Jet ventilation has also been safely used in obese patients with a BMI ≥ 40 at the time of surgery [96].

In a prospective RCT, Fadaizadeh et al. compared the safety and efficacy of using either an LMA with standard positive pressure ventilation or rigid bronchoscopy and jet ventilation [98]. A total of 83 patients were recruited, 45 in the “LMA” group and 38 in the “rigid” group. Both oxygenation (*p* = 0.028) and hemodynamic stability were better maintained in the LMA group. Typical ventilation was accomplished with the following settings in the LMA group: tidal volume: 10 cc kg^−1^; respiratory rate: 12/m; FiO_2_: 100%; O_2_ flow: 5 lit. The jet ventilator settings in the rigid group were as follows frequency: 150–200 R min^−1^; PIP: 25; FiO_2_: 100%; driving pressure: 2.5–3 bar; minute ventilation: 15–20. Other researchers have published their experience with the use of LMA in interventional bronchoscopic procedures [99,100].

In conclusion, tracheal lesions can be treated under general anesthesia, typically with the TIVA technique, and effective ventilation can be established with either an LMA or using a jet ventilator. Inadequate ventilation, pneumothorax, subcutaneous emphysema, excessive gastric inflation, severe arrhythmias, aspiration, and cardiac arrest are some of the complications.

## 9. Preparing for Possible Bleeding in Airways and Potential Interventions

Among the many complications described during bronchoscopic procedures, bleeding remains the most severe and is potentially fatal. The Delphi consensus statement from the Nashville working group proposed a standardized definition to quantify bleeding after transbronchial lung biopsy [101]. They described four grades. If blood needs to be suctioned for less than a minute, it is considered grade 1; suctioning for more than a minute or repeated wedging for persistent bleeding and the application of cold saline, dilute vasopressors, or thrombin is grade 2; the use of selective lung/lobe ETT or balloon blockers for less than 20 min or premature procedure interruption is described as grade 3; and finally, grade 4 is described as persistent selective intubation for >20 min, new admission to the ICU, packed red cell transfusion, or the need for bronchial artery embolization or resuscitation.

In a retrospective chart review involving 45,734 patients who underwent diagnostic or therapeutic bronchoscopies, the rate of severe complications was 0.85%, with a mortality of 0.01% [102]. Depending on the degree of bleeding, their management approach included only suctioning, pressure via an inflated Fogarty catheter/bronchial blocker, intravenous pituitrin (a pituitary extract containing vasopressin), and, in some cases, transfusion of blood products or interventional or surgical therapy. Severe bleeding is mainly seen during bronchial biopsies, especially after conventional or cryoprobe-assisted transbronchial biopsy [103]. Therapeutic bronchoscopic procedures carry a higher rate of bleeding (37.76%), especially with multiple biopsies, and about 1.63% suffer severe bleeding [102]. Biopsying the left upper lobe and bronchus intermedius carries higher risk of bleeding compared to other locations.

Li et al. analyzed a total of 1482 bronchoscopy procedures with central airway obstruction (CAO) needing interventional bronchoscopy and, sometimes, multiple rounds of treatment [104]. The first treatment carried a significantly higher risk of bleeding than repeat treatments. In addition, procedures performed under general anesthesia posed less bleeding risk. Those with pulmonary hypertension (as evidenced by echocardiography or chest CT) also carry a higher risk of bleeding [105]. In this retrospective study, medical records of 314 consecutive patients who underwent endobronchial ultrasound-guided transbronchial biopsy using a guide sheath, as well as echocardiography and chest CT, were analyzed. In total, 35 patients (11.1%) had documented bleeding; in the subgroup of patients with pulmonary hypertension, the risk of bleeding was 29.4% (5/17).

Patients with thrombocytopenia present a higher risk of bleeding, and platelet counts of 20,000 to 50,000 are considered as safe [106]. Although lower counts might be acceptable for routine airway inspection with bronchoalveolar lavage, procedures such as needle biopsy, transbronchial biopsy, or cryo-biopsy should be treated in a separate category with higher thresholds.

It is important to have a standardized approach in terms of bleeding management. Our approach at Jefferson University Hospital, Philadelphia, PA, USA, is similar to established approaches such as those described by Bernasconi et al. [103]. The bronchoscope could be wedged into the segment of the lung from where bleeding is coming from. In addition to the reverse Trendelenburg position (head high), the patient is turned to one side so that the bleeding side is dependent. This prevents soiling of the normal lung with blood. Cold saline and vasoconstrictors such as dilute epinephrine are instilled. Other options are applying pressure via a device such as a Fogarty balloon catheter. Electrocautery and argon plasma coagulation should be always immediately available when dealing with endobronchial lesions. Fluid resuscitation and administration of intravenous vasopressors/inotropes may be required. Some of the last resorts are surgery that carry significant morbidity and mortality and bronchial artery embolization.

Endobronchial instillation of an absorbable gelatin and thrombin slurry was used to secure hemostasis in thirteen patients when standard bronchoscopic measures like cold saline, epinephrine, and, in some cases, balloon occlusion failed [107]. The slurry could be delivered through the working channel of the bronchoscope or through the distal port available in some bronchial blockers.

## 10. Newer Sedatives in Bronchoscopy

Although the popularity of conscious sedation has fallen significantly in US practice, it remains a viable and often only the option in many parts of the world. In the USA, the use of conscious sedation is institutional and case/procedure-dependent. In our hospital, certain procedures such as bronchoalveolar lavage and the insertion of a PleurX catheter are routinely performed under conscious sedation. Although midazolam (a benzodiazepine) and fentanyl remain the main components of conscious sedation, two new drugs, remimazolam and dexmedetomidine, are gaining traction among bronchoscopists.

### 10.1. Remimazolam

This unique benzodiazepine was first synthesized by Glaxo Wellcome in 1990s. Since then, it has changed many hands and is currently marketed by Eagle Pharmaceuticals, Inc., in the USA. Described as a drug looking for an indication, there is no dearth of information on its potential role and utility, including for flexible bronchoscopy [108,109,110,111,112,113].

In terms of its actions, remimazolam is no different than its parent compound midazolam. It is a short-acting benzodiazepine with a rapid onset of effect and relatively short duration. The added benefit of remimazolam is its rapid offset of clinical effects, especially when used for short procedures such as bronchoalveolar lavage because of its unique elimination. It undergoes organ-independent metabolism to inactive compounds, and as a result, it is safe in patients with liver and kidney dysfunction. Additionally, its effects are easily reversible with flumazenil. Studies have reported utility of remimazolam both during rigid and flexible bronchoscopy.

In a prospective randomized controlled trial, Pan et al. (2022) compared remimazolam-flumazenil (Group R) and propofol (Group P) for rigid bronchoscopy [114]. A total of 34 patients were enrolled in this study. They found that Group R recovered from anesthesia much faster than Group P (140 ± 52 vs. 374 ± 195 s). It should be noted that, as of now, there is no agent that can reverse the effects of propofol. Patients in both groups were paralyzed with rocuronium. Re-sedation after flumazenil reversal is a possibility and needs to be looked out for [115].

In a prospective, double-blind, randomized, multicenter, parallel group trial that was performed at 30 US sites, remimazolam was administered under the supervision of a pulmonologist for flexible bronchoscopy with a success rate of 80.6%, while the success rates were 4.8% in the placebo arm and 32.9% in the midazolam arm [116]. In this study, the efficacy and safety of remimazolam for sedation during flexible bronchoscopy were compared with placebo and open-label midazolam. In another single-center, randomized controlled study, 51 patients were assigned to the midazolam group and 49 to the remimazolam group [117]. These patients underwent flexible bronchoscopy, and local anesthesia was also used to suppress coughing. While the physician satisfaction and willingness to repeat the procedure were similar in both groups, the remimazolam group exhibited a shorter time to reach peak sedation and time from the end of the procedure to full alertness. Similar results are reported in meta-analyses [118,119].

### 10.2. Dexmedetomidine

Dexmedetomidine is a selective α-2 adrenoceptor agonist that displays sedative, analgesic, anxiolytic, sympatholytic, and opioid-sparing properties [120]. Anesthesiologists have used it for decades as a sedative and adjuvant with other anesthetics and analgesics. Uniquely, it facilitates the transition from awake to sleepy to awake states, and this property is useful in procedures such as awake craniotomy [121,122]. It produces minimal respiratory depression, and this quality is useful in providing safe conscious sedation for flexible bronchoscopy. Its slow onset of clinical effect along with bradycardia and hypotension are some of the common drawbacks.

Wu et al. (2020) performed a retrospective chart review of all patients undergoing flexible bronchoscopy with moderate sedation [123]. Compared to those who received midazolam-propofol-fentanyl, patients sedated with dexmedetomidine-propofol-fentanyl showed higher safety with fewer procedural disruptions caused by cough or body movement. Dexmedetomidine was administered as a bolus of 0.7 μg/kg dexmedetomidine for 10 min, followed by a maintenance dose of 0.07 μg/kg/h. However, all patients were also administered 2 mL of 2% lidocaine and suctioned as needed to limit coughing. If the cough persisted or sedation was associated with body movements, 25–50 μg fentanyl was administered.

In a randomized controlled trial, Pertzov et al. (2022) tracked the number of desaturation events (primary outcome) and transcutaneous carbon dioxide (TcPCO_2_), hemodynamic adverse events, and physician and patient satisfaction (secondary events) [124]. After receiving a loading dose of fentanyl 1 mcg/kg and midazolam 1 mg, patients in the dexmedetomidine group were given a loading dose of 1 mcg/kg over 15 min followed by a continuous intravenous infusion at a rate of 0.5 mcg/kg/h, while the propofol group received 0.5–1 mg/kg for induction over 1 min followed by a maintenance infusion in a dose of 100–200 mcg/kg/min. They did not find any differences in oxygen saturation and cPCO_2_ levels between the two groups. However, the dexmedetomidine group required a significantly higher number of rescue boluses due to inadequate sedation and was associated with a higher rate of adverse events.

When used as a sedative along with local anesthesia, dexmedetomidine provided better patient cooperation and comfort [125]. In a meta-analysis, Guo et al. (2023) found that dexmedetomidine reduces the incidence of hypoxemia and tachycardia during bronchoscopy but is more likely to provoke bradycardia [126].

Dexmedetomidine is also used as a nebulizer. In this form, it increases patient comfort, reduces cough, improves tolerance, and is associated with shorter recovery time compared to the intravenous route [127]. In addition, it can relieve bronchospasm. In a double-blind randomized controlled trial (nebulized dexmedetomidine vs. nebulized saline) involving 100 patients, the nebulized dexmedetomidine group had limited efficacy in terms of reducing coughing compared to nebulized saline [128]. The procedures were mainly bronchoalveolar lavage and diagnostic bronchoscopy. In yet another study, Grover et al. (2024) demonstrated that nebulized dexmedetomidine has insignificant topical action in reducing cough episodes when compared to normal saline in diagnostic flexible bronchoscopy [129].

A summary of the anesthetic management procedures discussed here can be found in Table 1.

## 11. Conclusions

We have attempted to provide a concise description of both the anesthesia and procedural aspects of some of the newer bronchoscopic procedures. Interventional pulmonology is being recognized more and more as a distinct specialty, and additional therapeutic procedures such as LASER cautery, argon plasma coagulation, and photodynamic therapy are increasingly performed, in addition to rigid bronchoscopy. As an indispensable team member, the anesthesiologist plays a crucial role. In some institutions, medical thoracoscopy is performed with general anesthesia along with single lung ventilation, but usually, it is performed under moderate sedation.

Anesthesia requirements of electromagnetic navigational bronchoscopy are unique and aim to limit the development of atelectasis to preserve lung volumes. Deployment of endobronchial valves is associated with a high incidence of pneumothorax, and this is inevitable. Bronchial thermoplasty might be becoming less popular than it was. Among the newer sedatives, remimazolam is showing some promise, while dexmedetomidine is unlikely to change the landscape of bronchoscopy conscious sedation.

## Figures and Tables

**Table 1:** Summary of some advanced bronchoscopic procedures and their anesthesia management discussed in the current review.

Procedure	Indication and Preprocedural Factors	Anesthesia Requirements	Post-Procedure Factors
Navigational Bronchoscopy	Biopsying suspicious lesions. Patients are typically elderly with a history of smoking.	General anesthesia, usually TIVA with muscle relaxant. Larger than normal ETT (8.5) Avoid atelectasis without comprising safe oxygenation (SpO_2_ about 94%). Avoid 100% O_2_ even for induction. Air O_2_ mixture for maintenance. ETT position as guided by the bronchoscopist. Use appropriate ventilatory strategy to prevent atelectasis such as VESPA or LNVP (see text for details). Avoid barotrauma and hemodynamic instability during recruitment maneuvers.	Pneumothorax (3.4% to 9.8%). Respiratory failure and difficulty in extubation (rare).
Endobronchial valve placement	For lung volume reduction in selected cases of severe COPD	General anesthesia, TIVA with muscle relaxant. Larger than normal ETT (8.5) Hypotension to be expected and often requires phenylephrine support.	Pneumothorax is common, including tension pneumothorax. Incidence is reported as 4.2–34.4% (more often under GA than sedation). COPD exacerbation.
Bronchial thermoplasty	Indicated in selected patients with stable asthma without active respiratory tract infection. Should not have exacerbation of asthma for 2 weeks. Before BT, COPD to be excluded. Expect patients to have received steroids before procedure.	General anesthesia, glycopyrrolate for its antisialogogue properties, TIVA with muscle relaxant, ETT or an LMA.	Bronchospasm during or after bronchoscopy. Laryngospasm, atelectasis due to fibrin plugs. Exacerbation of asthma. Lower respiratory tract infections. Bronchial artery pseudoaneurysms.
